# Development and validation of a pregnancy symptoms inventory

**DOI:** 10.1186/1471-2393-13-3

**Published:** 2013-01-16

**Authors:** Katie F Foxcroft, Leonie K Callaway, Nuala M Byrne, Joan Webster

**Affiliations:** 1 Department of Internal Medicine, Royal Brisbane and Women’s Hospital, Brisbane, Australia; 2 Royal Brisbane and Women’s Hospital and School of Medicine, University of Queensland, Brisbane, Australia; 3 School of Human Movement Studies and Institute of Health and Biomedical Innovation, Queensland University of Technology, Brisbane, Australia; 4 Centre for Clinical Nursing, Royal Brisbane and Women’s Hospital, Brisbane, Australia

**Keywords:** Checklist, Inventory, Pregnancy symptoms, Questionnaire, Survey

## Abstract

**Background:**

Physical symptoms are common in pregnancy and are predominantly associated with normal physiological changes. These symptoms have a social and economic cost, leading to absenteeism from work and additional medical interventions. There is currently no simple method for identifying common pregnancy related problems in the antenatal period. A validated tool, for use by pregnancy care providers would be useful. The aim of this study was to develop and validate a Pregnancy Symptoms Inventory for use by health professionals.

**Methods:**

A list of symptoms was generated via expert consultation with health professionals. Focus groups were conducted with pregnant women. The inventory was tested for face validity and piloted for readability and comprehension. For test-re-test reliability, the tool was administered to the same women 2 to 3 days apart. Finally, midwives trialled the inventory for 1 month and rated its usefulness on a 10cm visual analogue scale (VAS).

**Results:**

A 41-item Likert inventory assessing how often symptoms occurred and what effect they had, was developed. Individual item test re-test reliability was between .51 to 1, the majority (34 items) scoring ≥0.70. The top four “often” reported symptoms were urinary frequency (52.2%), tiredness (45.5%), poor sleep (27.5%) and back pain (19.5%). Among the women surveyed, 16.2% claimed to sometimes or often be incontinent. Referrals to the incontinence nurse increased > 8 fold during the study period.

**Conclusions:**

The PSI provides a comprehensive inventory of pregnancy related symptoms, with a mechanism for assessing their effect on function. It was robustly developed, with good test re-test reliability, face validity, comprehension and readability. This provides a validated tool for assessing the impact of interventions in pregnancy.

## Background

Physical symptoms are common in pregnancy and are predominantly associated with normal physiological changes that occur during this time. Much of the literature reports on one or two specific symptoms but does not examine the range of potential symptoms possible during this time [1,2]. The need for such an instrument became apparent when designing our pregnancy intervention study [3]. Lifestyle and other interventions during pregnancy have the potential to alter the frequency and severity of the full range of pregnancy symptoms, such as nausea, back pain, incontinence, quality of sleep, mood or libido. We identified that there is no validated way of assessing the impact of pregnancy interventions on the wide range of pregnancy symptoms that women experience. Therefore, in this study we outline our approach to the development and testing of a valid and robust tool to assess pregnancy symptoms.

A review of the available literature identified a variety of pregnancy symptoms. Thirty-eight discrete symptoms have been described [4], with five being reported most frequently. These are: frequency of micturition (passing urine), fatigue, pelvic pressure, insomnia and lower backache. Although many studies have investigated a small number of pregnancy symptoms [5-7] such symptoms are not always experienced in isolation. However, the interaction of symptoms, such as back and pelvic pain causing sleep disturbance or sleep disturbance causing fatigue are not well explored. Psychosocial variables also impact on prevalence and frequency of pregnancy symptoms [8]. Many symptom specific instruments exist such as the McGill Nausea Questionnaire [9], The Fatigue System Checklist (FSC) [10], Roland-Morris Disability Questionnaire [11], ICIQ-SF (International Consultation on Incontinence Questionnaire Short Form) [12] and the Epworth Sleepiness Scale (ESS) [13]. While these instruments may be helpful in identifying particular symptoms they do not allow the examination of all potential symptoms and it is onerous to ask patients to complete multiple questionnaires.

If inventories are to be useful, they should undergo the same rigorous reliability and validity checks as other instruments prior to implementation. The aim of the project was to develop and validate a Pregnancy Symptoms Inventory (PSI) as a validated research tool which could be used to assess a range of pregnancy symptoms, and determine the impact those symptoms have on quality of life.

## Methods and results

### Development

**Figure 1:**
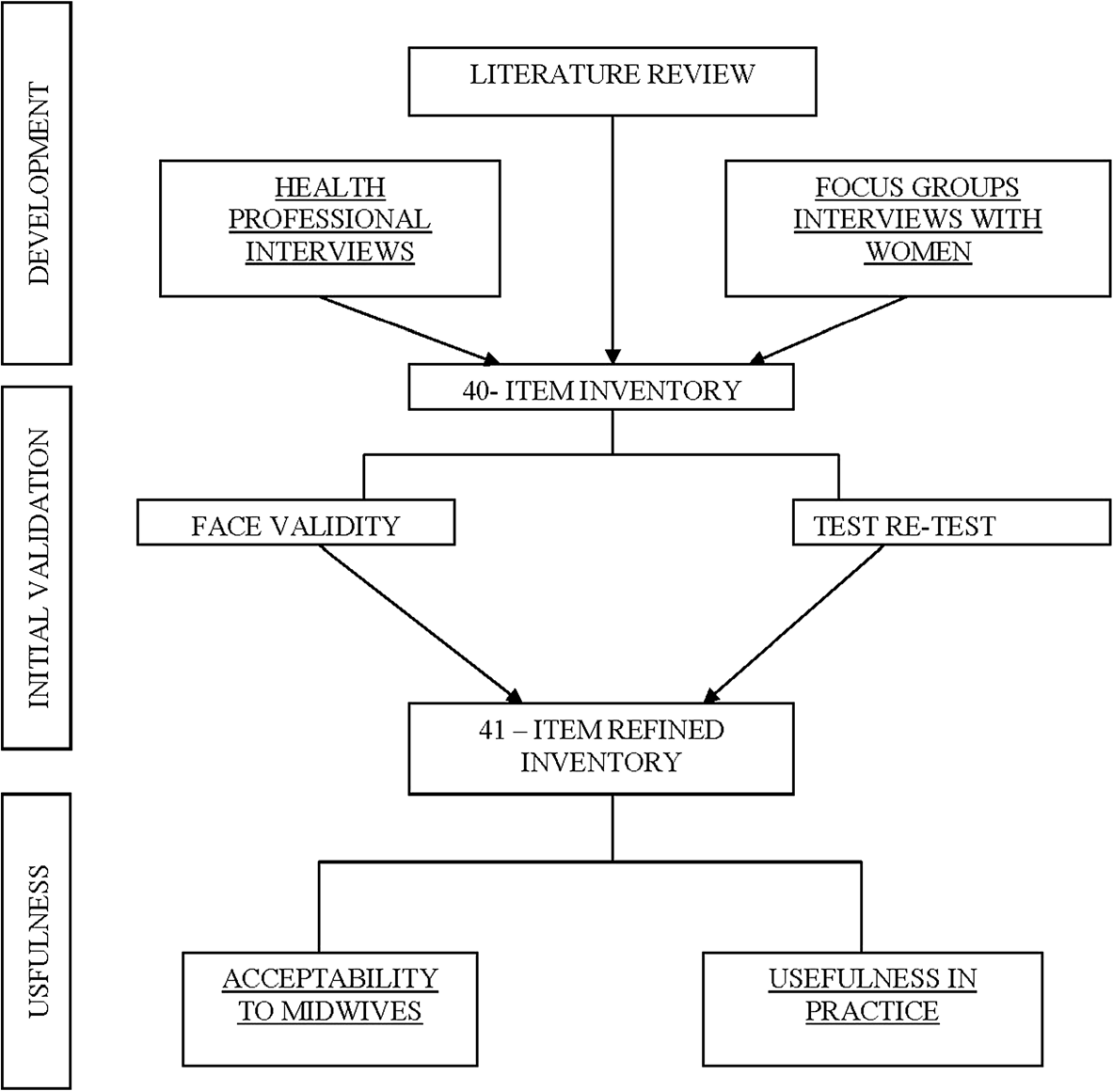
Framework for the development and testing of the PSI.

This study was conducted at The Royal Brisbane and Women’s Hospital (RBWH), a large tertiary referral hospital. Approximately 5000 pregnant women per year attend the Maternity Outpatient Department (MOPD). A mixed methods design was used; Figure 1 shows the framework for the development and testing of the instrument. Inventory development and testing included the use of interviews, surveys and focus group methods. Participants were medical and midwifery staff employed by RBWH who assisted in the development of the inventory and pregnant women who were involved in its refinement and testing. Due to the use of mixed methods in this study, strategies used for data analysis are reported by phase. All quantitative data was analysed using the statistical package SPSS version 16. Ethics approval was granted by the RBWH ethics committee.

#### Phase 1 (Expert consultation)

A group of seven health professionals (doctors and midwives) working in the MOPD were interviewed and asked to name all the commonly occurring symptoms of pregnancy reported by patients. The symptoms described by each person were kept in a log, to be reviewed and categorized after all the experts had been interviewed.

##### Results

During this phase 40 items were produced. The items mentioned most frequently were nausea, tiredness and sore breasts.

#### Phase 2 (Focus groups)

Pregnant women who were able to provide written informed consent and who had booked in to the MOPD for their obstetric care were included.

Focus groups consisted of three groups of 5–6 pregnant participants (Group 1 first trimester up to 12 weeks gestation; Group 2 second trimester 12 to 28 weeks gestation Group 3 third trimester 28 to 40 weeks gestation). The groups were divided by trimester as some symptoms such as nausea are more prevalent in the first trimester whilst back pain and sleeping problems are more prevalent in the last trimester. The conversation was audio taped and transcribed verbatim for analysis. The women’s terminology formed a basis for the wording of the items to ensure symptoms were clearly understood.

##### Results

Twenty symptoms were mentioned in focus groups, nineteen of which had also been mentioned by the experts; the additional item identified in the focus group sessions was vivid dreams. A 41- item Likert scale inventory was formulated via expert consultation and focus groups.

### Initial validation

Responses from interviews with professionals and focus groups interviews were combined to develop the Pregnancy Symptoms Inventory (PSI). Items were categorized by body part or system using terminology which is understood by lay people e.g. skin/hair, aches and pains, sleep etc.

The reliability, validity and usefulness of the inventory were tested in a number of ways.

#### Face validity

Initially, the entire inventory was reviewed by experts to test face validity. Two groups of 5–9 midwives were asked to comment and give feedback on the symptoms listed, language used and the positioning of symptoms on the inventory. All responses from the midwives were noted so that adjustments could be made to better clarify the inventory.

The tool was designed to be easily read and understood and was limited to one 2-sided page so it was quick and easy to fill out. The format allowed for pregnant women to add additional symptoms and the midwives to add comments and record their actions on the second page.

The Inventory was then piloted on a group of ten pregnant women of mixed ethnicity to test readability and comprehension.

##### Results

Three terms were not understood: Chloasma, Palpitations and Vaginal Varicose Vein. These words were changed to “Brownish marks on face”, “Heart Palpitations” and “Painful Veins in Vagina”.

#### Test re-test

The Inventory was completed by 20 women twice, 2–3 days apart to assess for test-re-test reliability. The first group of 20 women completed the first questionnaire at MOPD and the second test was completed at home 2–3 days later. A second group of 20 women were sent their first questionnaire to complete at home before a planned visit and were then asked to repeat the inventory while waiting for their planned appointment.

This was done to overcome a potential bias that suggests that answers may vary, depending on the environment in which the survey is administered [14].

##### Results

The test re test reliability was between .51 to 1, the majority (34 items) scoring ≥0.70.

### Usefulness

MOPD Midwives were educated about the use of the PSI at their routine lunch meetings and on a one-on-one basis. Women attending a follow up visit were asked to fill in the PSI and hand it to their midwife to peruse and assess. Any problems could be discussed and referred if necessary to other HCPs. Midwives used the PSI for one month and recorded any referrals on the form. After the one month trial had ended the midwives were asked to rate usefulness of the inventory (which was anonymous) on a10cm visual analogue scale. The scale ranged from “Not useful at all” to “Very useful”. After rating the usefulness quantitatively, midwives were also asked if using the instrument had prompted them to act or refer the woman. They were also asked to comment on any other aspect of the inventory using an open-ended format.

Because midwives responses to usefulness of the inventory on the 10 cm visual analogue scale were not normally distributed results were analysed using the median and range. Comments on the usefulness of the inventory were reported verbatim.

#### Practical use (Administration of the inventory)

**Figure 2:**
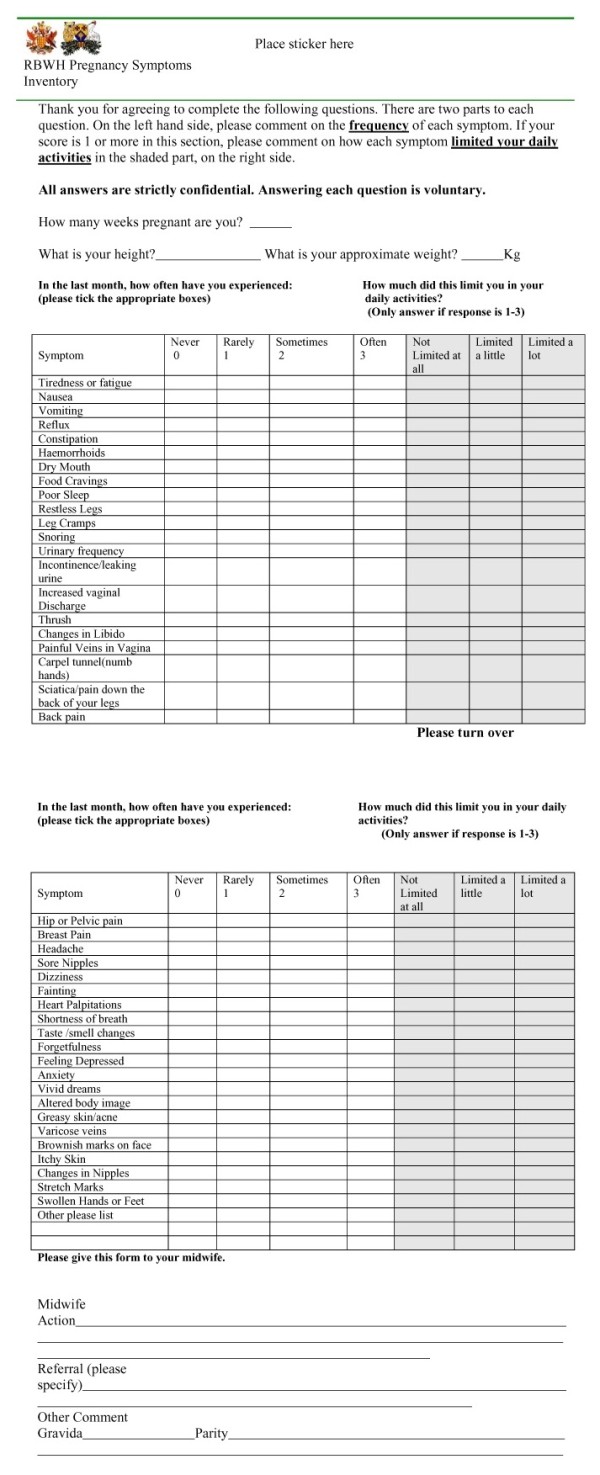
Pregnancy symptoms inventory.

Women attending the MOPD were asked to list any symptoms that had occurred in the previous month and rate them on a 4-point Likert scale according to frequency “never”, “rarely”, “sometimes”, “often”. They were then asked to rate using a 3-point Likert scale, how the symptom affected their activities of daily living as “not limited at all”, “limited a little” or “limited a lot”. See Figure 2. The women filled in their height, weight and number of weeks pregnant at the top of the inventory. The inventory was distributed when women presented at the clinic. Women completed the inventory while they waited, which took 5–7 minutes. This then enabled the midwife to quickly peruse the inventory, discuss any problems and make a comment in the “Midwife action”, “Referral” (please specify) or “comment sections” as to what actions, if any, she had taken.

Frequencies were determined by the number of those women experiencing a particular symptom in the previous month. Demographic data and incidence and severity of each of the symptoms were calculated using means and standard deviations (SD) for continuous data (e.g. age, gestation).

##### Results

The inventory was given to 211 women attending routine midwifery appointments for antenatal care. Responses were received for all 211 women. The mean age was 28.9 years (SD 6.16; range 15 – 44 years). The mean gestation was 23.06 weeks (SD 7.46; range 12–42 weeks). None of the women surveyed were in the first trimester of their pregnancy. This was because women do not present to MOPD for their first visit until 18 to 20 weeks unless they have a specific medical need and are then seen by specialist doctors not midwives for these visits. The majority (153) were in their middle trimester (≥12–28 weeks) and 52 were in their third trimester (≥28–42 weeks). The mean Body Mass Index (BMI) for respondents was 26.8 (SD 5.89).

Midwives trialled the inventory for 1 month and rated its usefulness on a 10 cm visual analogue scale (VAS).

The majority of midwives (7 out of 10) rated the usefulness at >7 and 4 rated it 10 out of 10. The median VAS score was 8.4 (range .9 to 10). Midwives found that the PSI alerted them to significant symptoms, and after further exploration, this prompted a referral for a specific need. Comments indicated that the tool was generally well accepted and that it had the potential for improving practice.

**Figure 3:**
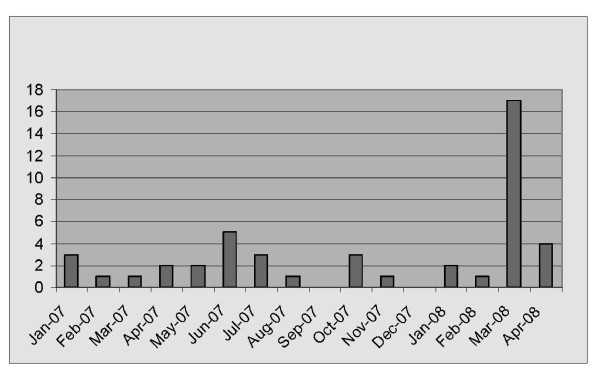
Incontinence referrals from maternity outpatients.

To assess outcome validity of the PSI, we assessed whether this triggered midwives to make referrals regarding symptoms identified. As a result, 35 women were referred to a physiotherapist; 13 women were referred to the continence nurse; eleven were referred to the mental health nurse. The continence nurse is the only specialist who keeps accurate records of all referrals she receives from the MOPD. Consequently, she was able to provide a snap shot of referrals made during the 12-month period in which the trial was conducted. The trial of the inventory ran from March 3rd 2008 to 9th April 2008 in the MOPD. As can be seen in Figure 3, there was a notable increase in the number of referrals to the continence nurse in the month of March 2008 (from an average of 1.8/month for the year up to 17 referrals in March) when the pregnancy symptoms inventory was being trialled. This represents more than an eight-fold increase in the number of referrals. The effect of the inventory on referrals is further demonstrated by a return to normal referral patterns once the trial finished.

The top four “often” reported symptoms were urinary frequency, tiredness, poor sleep and back pain, as outlined in Table 1. These were similar to the top four symptoms that women described as “Limit a Lot” their activities of daily living (ADLs); these were back pain, tiredness, poor sleep, and nausea. The total number responding to questions on “Limit” is less as only those who had experienced that symptom were required to give an answer. Whilst some of the symptoms had occurred ‘often’ a number of symptoms reported less frequently had the potential to adversely affect the women’s ADLs. For example, incontinence was reported to occur “often” by only eight women. However, 34 reported it occurred sometimes or often and 16 said that this affected their ADLs. Similarly, Feeling depressed “often” was reported infrequently. However many more women said depression affected their ADLs. Poor sleep was reported by 58 women as occurring often however 96 women said that poor sleep affected their ADLs. Table 2 contains full details.

**Table 1:** **Prevalence of self**-**reported pregnancy symptoms reported often or sometimes**

**Frequency**	**N** = **Valid responses**	**Often**	**Sometimes**	**Total prevalence**
Urinary Frequency	209	52.20%	33%	85.20%
Tiredness	209	45.50%	41.50%	87%
Poor Sleep	211	27.50%	35.05%	62.55%
Back Pain	210	19.50%	40.50%	60.00%
Vaginal Discharge	205	17.60%	32.20%	49.80%
Forgetfulness	198	15.70%	39.90%	55.60%
Headache	200	14.50%	36%	50.50%
Vivid Dreams	201	13.90%	27.40%	41.30%
Taste Smell Changes	197	13.70%	20.30%	34.00%
Change in Nipples	196	13.30%	25%	38.80%
Nausea	207	12.60%	21.70%	34.30%
Change in Libido	197	11.20%	32%	43.20%
Hip Pelvic Pain	199	10.60%	23.10%	33.70%
Constipation	207	10.10%	23.10%	33.20%
Food Cravings	208	9.10%	28.40%	37.50%
Reflux	203	8.90%	20.70%	29.60%
Leg Cramps	210	8.60%	22.90%	31.50%
Dizziness	200	8.50%	19%	27.50%
Stretch Marks	193	7.80%	10.90%	18.70%
Greasy Skin Acne	199	7.50%	18.60%	26.10%
Restless Legs	208	7.20%	23.10%	30.30%
Dry Mouth	210	7.10%	25.70%	32.80%
Breast Pain	173	6.50%	25.50%	32.00%
Altered Body Image	190	6.30%	19.50%	25.80%
Vomiting	210	6.20%	11.40%	17.60%
Sore Nipples	199	6.00%	23.60%	29.60%
Shortness of Breath	201	5.00%	25.90%	30.90%
Itch	198	4.50%	17.70%	22.20%
Snoring	207	4.30%	10.60%	14.90%
Varicose Veins	194	4.10%	12.90%	17.00%
Incontinence	209	3.80%	12.40%	16.20%
Carpel Tunnel	208	3.40%	7.70%	11.10%
Sciatica	209	3.30%	14.40%	17.70%
Anxiety	200	3.00%	16.50%	19.50%
Chloasma	201	3.00%	3.00%	6.00%
Thrush	203	2.50%	6.40%	8.90%
Painful Vein in Vagina	204	2.50%	2.90%	5.40%
Haemorrhoids	203	1.50%	4.40%	5.90%
Feeling Depressed	201	1.50%	20.40%	21.90%
Heart Palpitations	197	1.00%	9.10%	10.10%
Fainting	198	0.00%	2.50%	2.50%

As the number of women responding to questions varied, the denominator is displayed for each symptom (N).

**Table 2:** **Prevalence of self**-**reported limitations to activities of daily living**

**Frequency**	**N**	**Limit a lot**	**Limit a little**	**Total prevalence**
Back pain	173	11.00%	41%	52.00%
Tiredness	173	9.20%	67.30%	76.30%
Poor Sleep	176	8.50%	46%	54.50%
Nausea	186	7%	23.10%	30.10%
Headache	167	6.60%	44.30%	50.90%
Hip Pelvic Pain	199	6.30%	22.20%	28.50%
Vomiting	194	5.20%	13.90%	19.10%
Forgetfulness	168	4.80%	29.80%	34.60%
Sciatica	195	4.10%	9.30%	13.40%
Urinary Frequency	173	2.90%	36.40%	39.30%
Change in Libido	175	2.90%	14.90%	17.80%
Altered body image	172	2.30%	8.10%	10.40%
SOB	177	2.30%	23.20%	25.50%
Dizziness	177	1.70%	26.20%	28.30%
Stretch Marks	174	1.70%	2.30%	4.00%
Food Cravings	183	1.60%	3.30%	4.90%
Leg Cramps	183	1.60%	15.30%	16.90%
Restless Legs	184	1.60%	12%	13.60%
Incontinence	194	1.50%	6.70%	8.20%
Change in Nipples	171	1.20%	4.70%	5.90%
Constipation	185	1.10%	10.30%	11.40%
Reflux	186	1.10%	14%	15.10%
Thrush	194	1.00%	3.10%	4.10%
Painful vein in Vag	200	1.00%	2.50%	3.50%
Vaginal Discharge	173	0.60%	6.90%	7.50%
Taste Smell changes	178	0.60%	15.20%	15.80%
Breast Pain	174	0.60%	8.00%	8.60%
Anxiety	200	0.60%	12.20%	12.80%
Feeling Depressed	175	0.60%	15.40%	16.00%
Greasy skin acne	182	0.50%	4.90%	5.40%
Dry Mouth	189	0.50%	2.60%	3.10%
Itch	183	0.50%	4.40%	4.90%
Snoring	188	0.50%	1.60%	2.10%
Varicose veins	183	0.50%	4.40%	4.90%
Carpel tunnel	204	0.50%	4.40%	4.90%
Vivid dreams	175	0.00%	4.60%	4.60%
Sore Nipples	174	0.00%	8.20%	8.20%
Chloasma	190	0.00%	0.50%	0.50%
Haemorrhoids	199	0.00%	1.00%	1.00%
Heart Palpitations	190	0.00%	6.30%	6.30%
Fainting	190	0.00%	2.10%	2.10%

As the number of women responding to questions varied, the denominator is displayed for each symptom (N). Women who did not experience a symptom did not answer limit question.

## Discussion

Compared with instruments measuring discrete symptoms, our comprehensive pregnancy symptoms inventory provides a useful way of assessing the range of pregnancy symptoms, and determining the impact those symptoms have on quality of life. Women found it simple to complete and midwives confirmed its usefulness.

The PSI was developed within a robust framework, addressing face validity, test-retest reliability, outcome validity and practical use. We believe we have developed a tool which allows the assessment of the number and severity of pregnancy symptoms women experience.

We designed this tool to fill a gap we identified while conducting a randomized clinical trial [3]. We could not identify any brief comprehensive assessment tool to examine the vast range of pregnancy related symptoms in their entirety, including an assessment of the effect these symptoms have on daily life. Our tool assesses the full spectrum of pregnancy symptoms, and provides a way of comparing the burden of pregnancy symptoms between two arms of a clinical trial. We believe the PSI will be useful for other researchers who require an outcome measure of pregnancy symptoms to assess the impact of lifestyle or other pregnancy interventions.

Our research suggests that the PSI might also have a role in clinical practice. We identified that when midwives were prompted by this tool, they undertook additional clinical assessment, which initiated referral to a range of health care providers. This resulted in additional care from physiotherapists, continence nurses and mental health professionals. These referrals may have been beneficial to the women, although our research was not designed to assess any improvement in women’s symptoms following such referrals. We believe the non threatening nature of our PSI may be particularly valuable in identifying women with issues that may be particularly sensitive or difficult to discuss, such as urinary incontinence. It has been estimated that total annual cost of urinary incontinence in women from the United States is around $12.4 billion [15]. Consequently, early identification of incontinence may lead to cost savings in this area. There is strong evidence that pelvic floor exercises performed during and after pregnancy can prevent urinary incontinence. One Australian study that investigated over 30,000 women in differing age groups found that one in three mid-age and older women experience leaking urine and that it was significantly associated with parity. This study suggested, as less than half of these women had sought help, that this issue should be brought up during their antenatal care [16]. Available evidence indicates, because incontinence is a somewhat embarrassing subject socially, that patients and medical practitioners alike are reticent about discussing it [17]. The PSI could be used to flag such problems; patients who are uncomfortable bringing up the topic may find it easier to “tick a box”, to alert their midwife or doctor that they are experiencing problems. Further, since the PSI has been designed to be used at various time points, it may be helpful in the early detection and treatment of pregnancy symptoms, which in turn may reduce absenteeism via early medical intervention. This is an area that would require further study.

Our PSI was developed in Australia, in a multicultural, English speaking cohort, and so it may not be transferrable to other populations. Further testing of the usefulness of the inventory in larger populations and in different settings is required to test the external validity of the instrument. It would also be useful to test the effectiveness of the PSI using a randomised control trial, to assess outcomes such as: frequency of symptom-related referrals; details of pathology tests ordered; information about other requested tests, such as X-rays and satisfaction with the prenatal booking in visit. Information about how to manage symptoms would also be a useful addition to the PSI.

While our process of PSI development was robust, the initial version missed the important symptom of swollen hands and feet. Once this was identified, all experts involved in the study agreed that this particular symptom should be included.

While we included focus groups with women from the first trimester, none of the participants who filled out our survey were in the first trimester. Therefore, any future research on the PSI should include women in their first trimester.

## Conclusion

In summary, we have developed a Pregnancy Symptoms Inventory that should be very useful in the research setting, and, subject to further research, may be a useful clinical tool.

## Competing interests

The authors declare that they have no competing interests.

## Authors' contributions

KFF: Has been involved in design of the study, data collection, analysis and drafting the manuscripts. NMB has been involved in interpretation of the data and revising the intellectual content. LKC has been involved in the design of the study and revision of the intellectual content. JW has been involved in the design of the study, provided oversight of the data collection and data analysis and revision of the intellectual content. All authors have read and approved the final manuscript.

## Authors' information

KFF: RN, RM, MAppSc (Research).

LKC: MBBS (Hons), FRACP, PhD.

NMB: BHMS, MAppSc, PhD.

JW: RN, RM, BA.

## Pre-publication history

The pre-publication history for this paper can be accessed here:

http://www.biomedcentral.com/1471-2393/13/3/prepub
